# A dynamic, ensemble learning approach to forecast dengue fever epidemic years in Brazil using weather and population susceptibility cycles

**DOI:** 10.1098/rsif.2020.1006

**Published:** 2021-06-16

**Authors:** Sarah F. McGough, Leonardo Clemente, J. Nathan Kutz, Mauricio Santillana

**Affiliations:** ^1^ Computational Health Informatics Program, Boston Children's Hospital, Boston, MA 02115, USA; ^2^ Harvard T.H. Chan School of Public Health, Harvard University, Boston, MA 02115, USA; ^3^ Tecnológico de Monterrey, 64849 Monterrey, Nuevo León, Mexico; ^4^ Department of Applied Mathematics, University of Washington, Seattle, WA 98195, USA; ^5^ Department of Pediatrics, Harvard Medical School, Harvard University, Boston, MA 02115, USA

**Keywords:** dengue, forecasting, ensemble

## Abstract

Transmission of dengue fever depends on a complex interplay of human, climate and mosquito dynamics, which often change in time and space. It is well known that its disease dynamics are highly influenced by multiple factors including population susceptibility to infection as well as by microclimates: small-area climatic conditions which create environments favourable for the breeding and survival of mosquitoes. Here, we present a novel machine learning dengue forecasting approach, which, dynamically in time and space, identifies local patterns in weather and population susceptibility to make epidemic predictions at the city level in Brazil, months ahead of the occurrence of disease outbreaks. Weather-based predictions are improved when information on population susceptibility is incorporated, indicating that immunity is an important predictor neglected by most dengue forecast models. Given the generalizability of our methodology to any location or input data, it may prove valuable for public health decision-making aimed at mitigating the effects of seasonal dengue outbreaks in locations globally.

## 1. Introduction

Owing to emerging sensor technologies and computational advances, the last decade has seen significant strides in the way data are generated and collected, resulting in large volumes of complex information known as ‘big data’. The recent availability of these data has opened up the possibility of new and complementary avenues for epidemic monitoring that leverage diverse data modalities such as satellite imagery [1,2], Internet search engine activity [3,4], social media [5], mobile phones [6,7], genomics [8,9] and disease surveillance databases [10,11]. This has opened up opportunities to posit and explore more hypotheses for characterizing the causes and outcomes of disease transmission, population behaviour, environmental conditions and other potential indicators of population health. Exploiting these relationships to generate reliable prospective forecasts would benefit health systems by allowing early mobilization of resources for the prevention of morbidities and deaths in the face of public health threats. A major challenge in disease forecasting is developing algorithms that can autonomously and continuously learn from these complex and ever-changing dynamical systems, uncovering patterns and signals with little human effort. Machine learning algorithms are ideally suited for such tasks. Indeed, they are having a profound impact across a wide range of application fields because of their ability to aid in learning and discovery.

One such complex system is the interplay of human, climate and mosquito dynamics that give rise to the transmission of mosquito-borne diseases such as dengue. Dengue fever, a viral mosquito-borne disease transmitted predominately by the *Aedes aegypti* and *Aedes albopictus* mosquitoes, infects an estimated 390 million people per year, with nearly half the world's population living at risk of infection [12]. The global burden of dengue has doubled every 10 years over the last three decades [13], and the disease is projected to expand its latitude range as global temperatures increase and create new suitable habitats for the *Aedes* mosquitoes among previously unexposed human populations [14]. Short-term climate conditions, particularly temperature and precipitation, can create favourable conditions for the breeding and survival of *Aedes* mosquitoes that may increase the transmission of the dengue fever virus in humans. Distinct ranges of temperature and precipitation have been observed to have an influence on the extrinsic incubation period [15,16], mosquito maturation rate [17], length of larval hatch time [18], survival rate [19] and biting rate [20]. However, the relationships that govern these parameters and give rise to dengue transmission are complex and dynamic, changing over time and across geographies. Moreover, multi-year cycles of dengue fever outbreaks, caused by one or more circulating dengue fever serotypes (DENV I, II, III, IV) and short-term immunity conferred after infection, add an important layer of complexity to prediction [21].

The dengue forecasting literature lacks a systematic, self-adaptive and generalizable framework capable of identifying weather and population susceptibility patterns that may be predictive of dengue fever outbreaks, particularly at the city level. Vector-borne diseases commonly exhibit spatial heterogeneity, a result of spatial variation in vector habitat, weather patterns and human control actions [22–25]. For developing forecast systems, this feature implies a trade-off between model consistency and spatial resolution. As a consequence, most studies to date focus on producing *ad hoc* predictions for a single location, ranging from the national to the city level [26–28], while others build and evaluate multiple modelling strategies per study site in efforts to manually identify relationships between weather patterns and dengue incidence over different geographies and temporal windows [29,30]. Both approaches highlight the difficulty in producing forecast models that are viable in diverse settings. By contrast, data-driven techniques demonstrate promise by learning from multi-scale, complex systems and automatically adapting to new information. A recent descriptive study showed the promise of a data-driven approach in identifying weather patterns with meaningful signals for dengue fever outbreaks [31]. Specifically, their data-driven strategy identified temperature and frequency of precipitation as key features in forecasting dengue outbreaks by extracting windowed time intervals for different cities that were highly predictive. Motivated by such learning algorithms, we build upon this data-driven strategy to build a richer, supervised forecasting algorithm.

## 2. Results

### 2.1. Exploiting weather signals to create a data-driven forecast system

**Figure 1.**
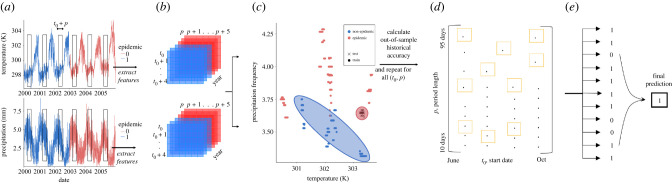
Ensemble forecast workflow. (*a*) To predict next year's epidemic status, we extract features from a daily time series of temperature (K) and precipitation (mm) over a defined (*t*_0_, *p*) time interval and for each year in the training period. (*b*) We produce an array of features corresponding to the mean value of temperature and precipitation over the (*t*_0_, *p*) interval and (*c*) train an SVM to classify next year's epidemic status. (*d*) This process is repeated for all 432 (*t*_0_, *p*) intervals, and the top 11 models are automatically selected to (*e*) contribute to a majority voting system based on historical out-of-sample accuracy.

We obtained data on both annual dengue fever cases (Brazilian Ministry of Health) for 2001–2017 and on daily temperature and precipitation (GMAO-NASA) for 2000–2016, for 20 dengue-endemic municipalities (figure 1; electronic supplementary material, table S1) in Brazil. Weather patterns were extracted and analysed across hundreds of partially overlapping time intervals collectively spanning the last seven months of a given year, a time period that typically precedes the onset of epidemic outbreaks in Brazil. Each of these patterns was then assessed for its ability to predict an outbreak year (defined as a year in which the number of cases exceeds 100 per 100 000 persons) for the subsequent year. Retrospective and fully out-of-sample forecasts, trained on a yearly expanding window, were produced for 10 years (2008–2017) and for each time interval using support vector machines (SVMs), a binary classifier. Every year, the time intervals with high historical predictive power were automatically selected and evaluated in the upcoming year to produce out-of-sample predictions for the subsequent dengue season (figure 1). An ensemble approach was then implemented to determine, in a completely out-of-sample fashion (using the first 4 years of out-of-sample predictions to inform ensemble model selection), the system's final prediction: whether a year would be epidemic or not for the next 6 years (2012–2017).

**Figure 2.**
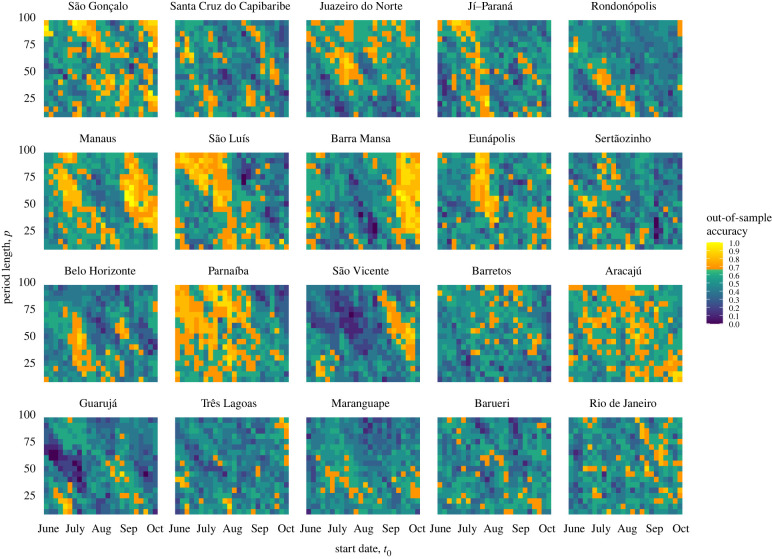
The 10 year (2008–2017) out-of-sample forecast accuracy (%) for each time window of temperature and precipitation, by the municipality. The *x*-axis (*t*_0_) indicates the start date of the time interval, and the *y*-axis (*p*) indicates the length of the time interval from which weather data were gathered (10–95 days). Models achieving at least 7/10 correct out-of-sample forecasts are shown in shades of yellow. Municipalities are ordered by decreasing ensemble prediction accuracy; that is, the proportion of years correctly forecast by the ensemble method over the years 2012–2017.

**Figure 3.**
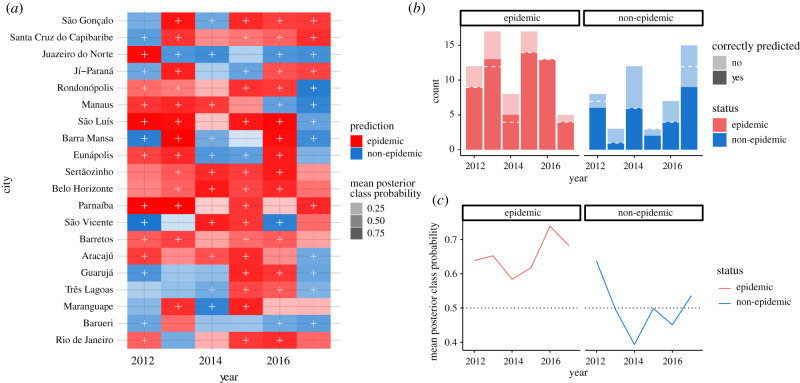
Weather-based prediction results for 120 municipality years. (*a*) Annual out-of-sample forecasts of outbreak status (epidemic/non-epidemic) for 20 Brazilian municipalities from 2012 to 2017, shaded by the mean posterior probability of the true outbreak status. Correct forecasts are indicated by a plus (+) sign, and cells with light shading indicate that the model predicted the class with low probability. Municipalities are ordered by decreasing ensemble prediction accuracy; that is, the proportion of years correctly forecast by the ensemble method over the years 2012–2017. (*b*) The number of total epidemic and non-epidemic years correctly forecast across 20 municipalities, by year. The dashed white line indicates the number correctly forecast after the incorporation of empirically observed dengue cycles. (*c*) The mean posterior class probability across municipalities, by year and epidemic status.

This system, which autonomously identifies and exploits the predictions of multiple time windows during the calendar year, makes it possible to identify temporally similar regions of highly predictive periods of the year preceding dengue outbreaks, here referred to as ‘weather signatures’. Weather signatures represent time windows across years that show strong influence (predictive power) on the incidence of dengue in a subsequent year. We observed that cities where our methodology led to higher prediction accuracy tended to have clear and robust weather signatures over the years, while cities where our approach was not strongly predictive did not exhibit consistent and robust weather patterns (figures 2 and 3*a*). Further, we observed that strong weather signatures in our sample of cities often corresponded with or preceded important alternating tropical seasons, such as rainy and dry seasons.

### 2.2. Weather-based forecasting performance

Using weather data (temperature and frequency of precipitation) alone to predict annual dengue outbreaks, our approach correctly forecast 81% of all epidemic years across 20 municipalities in Brazil between 2012 and 2017 (table 1, figure 3). For reference and as a baseline, the frequency of epidemic and non-epidemic years was 60% and 40%, thus a naive approach that predicts that all years are epidemic (the class majority) would achieve an overall accuracy of 60%. Our approach only identified 58% of non-epidemic years correctly. This resulted in an *overall accuracy* of approximately 72%. Our approach significantly exceeded *p* = 0.005, the predictive power of a naive predictor.

**Table 1.** Performance of weather-based out-of-sample forecasts across 120 municipality years in Brazil, with and without consideration for DENV susceptibility cycles.

evaluation metric	weather	weather + DENV cycle
accuracy	71.70%	75%
hit rate (sensitivity)	81%	78%
non-epidemic detection rate (specificity)	58%	71%
no-information rate	60%	60%
*P* (accuracy > no-information rate)	*p* = 0.005	*p* = 0.0004

### 2.3. Incorporating empirically observed dengue susceptibility cycles

The previously described weather-based ensemble approach ignores important factors that may influence the emergence of epidemic outbreaks from year to year, such as the population susceptibility to being infected with the virus. Specifically, endemic transmission of dengue fever is typically distinguished by periodic outbreak cycles of around 3–4 years. These outbreak cycles are thought to occur as a result of (i) an exhaustion of the susceptible population after an outbreak and (ii) short-term cross-immunity to other circulating DENV serotypes after infection [21], although the cycles can also be complicated by increased severity of a second infection [32]. Both factors result in a depletion of the population vulnerable to infection and act as barriers to subsequent outbreaks. Independent of climate variability over the years, we expect some preservation of these susceptibility cycles.

Inspired by this phenomenon, we implemented a data-driven hidden Markov model by empirically computing the frequency of transitioning between multiple sequences of epidemic and non-epidemic years (described in detail in the electronic supplementary material). Given the previously observed sequence of consecutive outbreak and non-outbreak years (dengue fever cycles), the Markov model computes the probability of the next year being an outbreak or a non-outbreak year. This acts as a proxy to dengue fever susceptibility in the population as it accounts for the cyclical nature of outbreaks that may be influenced by, for example, a depletion of the susceptible population following multiple years of high dengue activity. The approach is implemented as follows: if the weather-based approach makes a prediction with low probability, a decision rule is implemented to automatically override the weather-based prediction if the hidden Markov model (based on the pattern of consecutive outbreaks and non-outbreaks in years prior) predicts a more likely scenario. In this way, the ‘cycles’ of dengue fever outbreak susceptibility are incorporated into our otherwise agnostic weather-based approach.

### 2.4. Combining dengue cycles with weather patterns improves forecasts

Compared with the exclusively weather-based approach, incorporating these empirically observed dengue cycles into our system improved our ability to predict non-epidemic years by approximately 20% (specificity = 69%) and increased overall accuracy to 74.2% (table 1). Specifically, the additional decision rule replaced seven epidemic forecasts with non-epidemic forecasts, of which five were correct (figure 3*b*). The majority of these cases belonged to cities which had experienced three consecutive epidemic years leading up to the prediction.

Overall, the combined approach (weather-based plus dengue cycles) was dominantly driven by weather patterns and informed by the decision rule only in a few cases when historical data showed a very strong likelihood of either an epidemic or not epidemic year happening. Thus, the decision rule to favour the Markov model acts as an ‘expert opinion’ for situations in which there is clear evidence that a given predicted outbreak scenario (even if suggested by the weather patterns) is unlikely. Our specific finding—that the dengue cycles were used exclusively to overturn epidemic forecasts—suggests that while the weather conditions in those locations and years were identified to be conducive to an outbreak, there was stronger evidence that the population may have had low susceptibility to infection (thus avoiding an outbreak), based on multiple consecutive preceding years of high disease incidence.

### 2.5. Model performance by year

The success of our combined epidemic forecasts varied by year, reflecting the difficulty of forecasting disease activity relying only on weather patterns and the empirically extracted susceptibility cycles. During the last three years of the time series (2015–2017), epidemics were predicted by the weather-only models with at least 80% accuracy, with 100% of the 13 outbreaks in 2016 correctly forecast (figure 3*b,c*). Conversely, non-epidemic years during 2013–2014 were particularly difficult to predict, with only one-third and one-half of cities correctly forecasting non-epidemics for these years, respectively. The most successful non-epidemic predictions occurred in 2012, for which six out of eight non-epidemics (75%) were predicted correctly. Overall, 2015 and 2016 were the most successfully classified years, with 80% and 85% of municipalities correctly classified as epidemics or non-epidemics, respectively, while 2014 and 2017 were the most difficult years to predict, with 45% and 35% of municipalities misclassified, respectively.

Incorporating information on the dengue cycles helped detect an additional non-epidemic in 2012 and 2015, and an additional three non-epidemics in 2017 (figure 3*b*).

### 2.6. Quantifying the strength of predictions

Because our forecast system produces deterministic binary predictions (epidemic/non-epidemic year) using local-in-time SVM classifiers, a natural question is how to quantify the conviction (or confidence) of each prediction. It is important to note that the number of observations per city is small (*n* = 17), and, thus, a rigorous probabilistic approach to quantifying conditional probabilities of success is not feasible. However, in the interest of better communicating to public health officials the reliability of our predictions in a given location and time period, as well as identifying the determinants of success of our prediction system if one were to extend our predictive approach to new locations, we explored simple ways to characterize the accuracy and conviction of predictions. We did this based on both the historical performance of the selected ensemble generating the prediction and the performance of the weather-based classifiers themselves.

Our prediction system combines the output of a collection of local-in-time binary classifiers that use different time periods (characterized by an initial point in time, *t*_0_, and a window length, *p*), prior to the typical date of the onset of dengue outbreaks, as predictors. For each city and each year, the combination of these outputs is calculated using a voting system that only considers time windows that have consistently exhibited the highest historical out-of-sample prediction performance among all other time windows of the calendar year. In our framework, time windows are automatically selected into the forecasting ensemble if (i) their own historical out-of-sample performance is high and (ii) the historical performance of their calendar neighbours, that is, models using temporally nearby time windows as predictors, is high as well.

**Figure 4.**
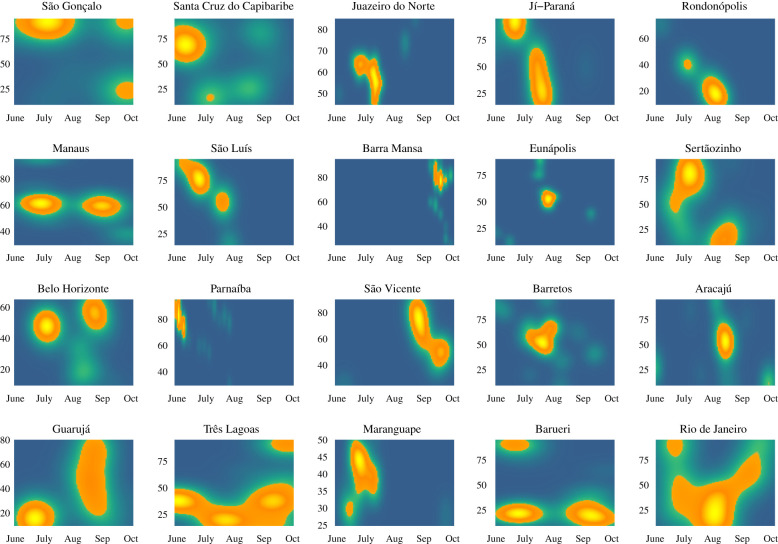
Periods of the year selected into the ensemble forecast model for 2012–2017, by the municipality. The *x*-axis (*t*_0_) indicates the start date of the time interval, and the *y*-axis (*p*) indicates the length of the time interval from which weather data were gathered (10–95 days). Municipalities with smaller and brighter yellow centres are those which exhibit the highest consistency in the predictive performance of weather patterns. Municipalities are ordered by decreasing ensemble prediction accuracy; that is, the proportion of years correctly forecast by the ensemble method over the years 2012–2017.

Consequently, we computed metrics of ensemble accuracy and strength (or confidence) by quantifying both of these elements. We found that, in cities where the predictive performance of our approach is highest (electronic supplementary material, figure S2), the successful individual classifiers that contribute to our final prediction use as input temporal regions that are clustered around one another (as shown in figure 4), suggesting that the presence of temporally consistent weather patterns can be thought of as an indicator of the success of our methodology.

It is important to note that models with high historical prediction performance may still lead to poor outcomes if the weather data for the year of (out-of-sample) forecast do not clearly belong to an epidemic or non-epidemic class, as learned by the individual classifiers, and/or if its weather patterns happen to ‘look like’ those appearing historically in the opposite class.

In order to further assess the individual strength or conviction of each individual classifier, we estimated whether the separability or difference between the two classes (epidemic versus non-epidemic) was well captured by the classifier by extracting calibrated posterior probabilities of each SVM model using Platt's scaling [33]. The posterior probability reflects the distance to the separation boundary distinguishing epidemic and non-epidemic years on the basis of weather. Thus, a higher probability represents how strongly the weather patterns of the prediction year aligned with those experienced by prior outbreak or non-outbreak years. We observed that, in general, the probabilities were moderately calibrated, i.e. roughly 80% of predictions made with 0.8 probability were epidemics (electronic supplementary material, figure S3); however, the small sample size (i.e. six out-of-sample years for each of the 20 cities) limits the ability to interpret this feature appropriately. We found that this measure of separability was not a particularly good indicator of accuracy; that is, our approach failed even in scenarios with high separability. Several factors may be driving this finding, including insufficient training data and the influence of factors beyond weather (e.g. sociodemographic characteristics, land use) on outbreaks; we elaborate further in the electronic supplementary material.

Both approaches to characterize the confidence of our predictions—quantifying ensemble strength and quantifying the separability of the data—highlight separate limitations of our modelling framework. First, we expect that both a greater variety of environmental variables (e.g. humidity, vegetation and standing water) and non-environmental variables (e.g. human activity and public health interventions) will contribute to more accurate predictions by considering broader factors that contribute to dengue fever activity in a given location. Second, the robustness of our predictions was limited by a short time series of annual information, which may not be sufficient to detect clear differences in epidemic and non-epidemic years on the basis of weather alone. Nonetheless, our reproducible modelling framework can easily be extended to accommodate additional predictors and longer time series, and thus we highlight these as limitations of only the present case study, with potential for improved performance in other data settings.

## 3. Discussion

Here, we have presented a novel approach to forecasting dengue fever outbreak years in Brazil at its smallest administrative unit, the city level, using a single, dynamic and flexible modelling framework that uses only two weather variables and historical information on yearly dengue activity. Our approach automatically learns from weather and population susceptibility patterns of any inputted yearly time series of dengue incidence and leverages the best historical predictions to generate an ensemble forecast. We find that complementing our weather-based statistical approach with observed 3–4 year cycles of dengue fever outbreaks (as a proxy for population susceptibility) is key for our models to achieve higher accuracy and improve substantially in predicting non-epidemic years. These forecasts may provide timely information on dengue fever activity to policymakers months ahead of outbreak seasons. Further, our entirely data-driven models show an ability to learn from complex relationships between dengue epidemics and climatic conditions and identify, in vastly different locations, potentially relevant weather patterns with likely biological significance. Importantly, these models can be immediately extended to other locations, requiring no location-specific manipulation or inputs aside from a globally available time series of daily temperature and precipitation as well as a complete yearly record of dengue incidence.

Using weather information only, our models seek to characterize and exploit the predictive ability of distinct weather patterns preceding outbreak years. Because our framework automatically identifies the time periods for which weather patterns produce strong signals, it was possible to identify temporal weather signatures in multiple locations with vastly different ecosystems and geographical locations. For this, we observed that cities with better overall prediction accuracy had stronger weather signatures, suggesting perhaps some biological consistency. For example, the southeastern municipality of Barra Mansa (five out of six ensemble years predicted correctly) exhibited strong signals from time windows spanning the first half of the city's rainy season, in October through December of each year. Further north, the hot, wet and humid municipality of Manaus (five out of six ensemble years predicted correctly), situated at the mouth of the Amazon, appeared to show two distinct weather signatures straddling the driest month of the year, August. These patterns, generated from 10 years of out-of-sample model predictions, suggest that, in different regions of Brazil, the weather may affect dengue transmission differently and at different times of the year. However, in locations where weather-based predictions were less successful, these signatures were not distinct; for instance, Rio de Janeiro (three out of six ensemble years predicted correctly) showed no clear temporal trend. In cities such as these, we might expect to see a lower influence of weather patterns on transmission than with other predictors (e.g. sociodemographics, policy, population behaviour, human land use, vector abundance). We did not find clear patterns by geography, population density or municipality size. We believe this work should catalyse important research both on the local influence of weather patterns on dengue outbreaks and on the extent to which other factors drive outbreaks in these locations. Moreover, this data-driven approach may help generate hypotheses on the relevance of multiple factors that may influence the dynamics of seasonal dengue outbreaks.

Even weather conditions that appear highly suitable for an outbreak (or none), based on historical information, may be challenged by other factors that limit (or encourage) transmission of dengue. A key strength of our approach is the incorporation of empirically observed information on dengue fever susceptibility cycles, to correct for potential short-term immunity that results from previous exposure to the dengue virus. We found that these susceptibility cycles were critical to the performance of models, particularly those which identified weather patterns suitable for a dengue outbreak in a year with potentially low population susceptibility to infection. For instance, this approach correctly identified three additional non-epidemics in 2017 compared with weather patterns alone, supporting the discourse on the unusually low dengue activity seen in Brazil in 2017 [34]. Still, our models missed half (6/12) of non-epidemics in 2014, which was predicted by experts to be a low transmission year because of the immunity provided by a large outbreak in 2013 with no changes in circulating DENV serotypes [34,35]. Thus, incorporating information on specific circulating serotypes could be used to better detect changes in population immunity and enhance our approach. Empirical and modelling-based seroprevalence studies may aid with this component, though this surveillance information is more challenging to routinely acquire [36]. Regardless, here we highlight the importance of incorporating mechanistic processes of disease transmission into data-driven approaches that may be otherwise blinded to them.

Our approach achieved an overall accuracy of 75%, which we believe is promising considering the difficulties in predicting the target. To put our results in context, we visited other benchmarks in the dengue prediction literature. While most dengue forecast models predict a continuous outcome such as total incidence (rendering comparisons of performance metrics not possible), we do find that dengue weather-based predictions achieve overall lower accuracy than other comparator models and achieve varied performance across distinct geographical regions, for example in the work of Lauer *et al.* [30] and Johansson *et al.* [29]. To the latter point, we find similarities with our work in that weather-based predictions performed well in some Brazilian municipalities, but not others. In another study that predicted a comparable binary outcome, weekly outbreak status, in Malaysian districts using weather information such as temperature and rainfall, the authors found an overall 70% accuracy using an SVM classifier [37], though noted that weather variables were not the most predictive in the model.

Because dengue transmission is driven by multiple complex socioecological and biological factors, we expect our models to capture only a portion of the epidemiological triangle. Here, we show the performance of two simple and relevant weather indicators of dengue fever, but the incorporation of additional weather features (i.e. humidity, vegetation and soil water absorption) combined with a feature selection step may lead to improved accuracy of forecasts, by considering more complex weather conditions preceding dengue outbreaks. However, in initial exploratory analysis, we did not find that other weather factors such as humidity or soil absorption outperformed temperature and precipitation alone, confirming the findings of [31,38] that factors other than temperature and precipitation may have little influence on dengue outbreaks. We also demonstrate the robustness of this approach by replicating the study using an alternative feature extraction method, singular value decomposition, with similar results (electronic supplementary material, figure S4). Nonetheless, we show that weather predictions fail in some cities, for example Rio de Janeiro (discussed above), where non-climatic factors may be influential in dengue outbreaks. For example, social factors including socioeconomic conditions [39], population mobility dynamics [40] and public health and infrastructure [41], as well as mosquito factors such as vector abundance [42], are known contributors to dengue transmission. These variables may contribute to a more complete understanding of dengue fever in Brazil. Our work shows that weather- and susceptibility-based models can contribute valuable information to larger ensemble approaches that leverage a collection of mobility, sociodemographic, epidemiological, climatic and biological information. Future work should explore the incorporation of these comprehensive data into a single modelling approach.

Our approach also demonstrates the feasibility (and limitations) of predicting in a ‘small data’ setting, wherein only 17 outcome data points were available in total for training and out-of-sample predictions (each representing annual outbreak status between 2001 and 2017). We chose a short training period (initial 7 years) to maximize the number of out-of-sample ensemble predictions, but ultimately it is difficult to establish strong climatic distinctions between outbreak and non-outbreak years in the data with so few samples. Thus, we anticipate improvement in performance for settings that have multiple decades of data, which would allow for longer training periods, improved separability in the data and more stable identification of dengue susceptibility cycles, all improving the quality, robustness and accuracy of predictions. In addition, where epidemiological data are available at finer temporal resolutions (e.g. weekly, monthly), this prediction problem could leverage more classical time-series approaches (such as SARIMA models) that incorporate adjustments for seasonality and trends, for example, as was done in [29]. Future studies should compare our approach with time-series-based methods wherever data are available to do so. Finally, our approach—which spans two decades and 20 locations—is limited by reporting heterogeneities in space and time. Brazil's centralized compulsory notification system, SINAN (Information System for Notifiable Diseases), has experienced software and reporting standards changes over the last two decades, giving rise to potential discrepancies in disease reporting at temporal change points. In addition, the case notification data in SINAN originate from data collected at health facilities via epidemiological disease surveillance reporting forms, and despite well-centralized reporting standards differences in reporting may exist between locations. However, dengue is a compulsory reportable disease in Brazil and receives a large number of reports nationwide each year (e.g. 1.7 million cases reported in 2015), and reporting is thought to accurately represent the overall trend of dengue in Brazil [43]. Because we further reduce the number of case reports to a binary outbreak status (epidemic/non-epidemic), our dependent variable may be less susceptible to these issues. Nonetheless, reporting heterogeneities are an inherent limitation to work like this.

Ultimately, this framework provides a simple, reproducible method of predicting dengue fever outbreak years in a wide range of locations. Given that the global and economic burden of dengue is placed at an estimated 390 million infections and US$8.9 billion per year [12,44], optimizing resource allocation for disease prevention is critical. However, control of the *Aedes* mosquito requires weeks or months before effects are seen on the vector population, so predicting dengue outbreaks up to several months before their onset is ideal. Our reproducible approach, which uses globally available data with the daily resolution, is intended to serve as a supervised learning framework to produce early outbreak warnings in any desired context, resulting in more efficient resource mobilization, budgeting and prevention campaigns. Moreover, the flexible approach can be extended to include other variables thought to be predictive of dengue outbreaks. Developing transparent early warning systems at the local level is emerging as a top global health priority, making our contribution both timely and impactful.

## 4. Material and methods

### 4.1. Study design

We developed a single, flexible modelling framework capable of identifying potentially useful weather patterns to predict dengue fever and used this to forecast annual outbreak status (epidemic/non-epidemic).

Our workflow, outlined in figure 1, combines elements from signal processing/spectral analysis, machine learning and ensemble modelling to achieve robust, data-driven epidemic forecasts that do not require any prior knowledge of the system (i.e. climatic influences on dengue transmission). Our research question is inherently one of time-series classification, to forecast epidemic versus non-epidemic years of dengue fever. The workflow begins with a time series of hourly and daily weather information, which serve as inputs to a collection of classifiers that contribute to ensemble-based epidemic predictions. Our approach can be described in five steps.

1. *Signal preprocessing*: for a time series of weather data, define time intervals of varying sizes (10–95 days across the last seven months of the calendar year) and use a windowing technique [31] to include information within several days of the interval. In contrast with [31], there are no deleterious effects due to missing temperature data since the data are acquired via satellite instead of ground measurements.
2. *Time-series feature extraction*: extract a simple summary measure for two weather variables with known influence on mosquito-borne disease dynamics, temperature and frequency of precipitation. Although more variables can be considered, they have little influence on the predictive power in comparison with the two selected [31].
3. *Independent model training and prediction*: train a collection of independent SVM classifiers on historical information from each unique time interval, and generate an out-of-sample epidemic prediction for the following year. Although SVM was used in [31], we provide here a richer out-of-sample prediction scheme for forecasting.
4. *Model selection*: choose the best 11 models, representing strongly predictive periods of the year preceding outbreaks, based on (i) historical out-of-sample prediction accuracy and (ii) out-of-sample performance of neighbouring time intervals.
5. *Ensemble prediction*: determine a final out-of-sample epidemic forecast by a majority vote of the selected top models.

To potentially enhance the performance of this exclusively weather-based approach, we implemented a *post hoc* step incorporating empirical information on 3- and 4-year dengue fever cycles as a proxy for population susceptibility to infection.

6. *Dengue cycles*: implement a decision rule governed by the second- and third-order Markov transition probabilities, reflecting the transition between consecutive sequences of epidemic and non-epidemic states

We applied our approach to 20 cities in Brazil spanning large geographical and population ranges (electronic supplementary material, figure S1 and table S1). We used as input a historical time series spanning 17 years and consisting of information on dengue case reports (number, annual) and two weather variables: 2 m air temperature (kelvin, daily) and precipitation (kg m^−2^, hourly). We describe data sources, acquisition and processing in the electronic supplementary material. After an initial training period of 7 years, we generated 10 years of out-of-sample epidemic predictions for each of the independent models using a 1 year expanding training window (step 2). We used the first 4 years of out-of-sample predictions to inform ensemble model selection (step 4) and produced ensemble-based predictions for the remaining 6 years (step 5).

### 4.2. Signal preprocessing

Using a daily time series of weather data to forecast dengue fever epidemic status requires identifying the most predictive period(s) of the calendar year during which weather information contains a strong signal for subsequent dengue fever outbreaks. In order to construct a single framework that can automatically identify important weather signals in multiple different locations with vastly different ecosystems and weather patterns, we allow the data to inform the choice of time intervals. Our algorithm achieves this by scanning over multiple, partially overlapping time intervals across the calendar year, and building hundreds of models on these different intervals in order to select those with the strongest signals.

Each time interval is defined by a start date, *t_*0*_*, between early June and late September, and a period length, *p*, of between 10 and 95 days. The combination of each (*t_*0*_*, *p*) produces multiple, partially overlapping intervals spanning the last seven months of the calendar year.

Borrowing from spectral analysis and wavelet decomposition, we use a windowing-inspired approach to better capture signals within the time intervals. Windowing is typically used to improve signal clarity, and here we apply a rectangular ‘range’ as described in [31] to incorporate the information in the days both within and around each time interval. We define a rectangle of 5 × 6, indicating that, for every defined (*t_*0*_*, *p*) time interval, the algorithm collects information from five consecutive start dates, *t_*0*_, t_*0*_ + *1*, …, t_*0*_ + *4**, spanning six consecutive period lengths, *p*, *p +* 1, …, *p +* 5. Each time interval and weather variable, then, is summarized by 30 data points, each capturing slightly different temporal slices from the time series. This process effectively adds a bit of redundant information to the model-building process—to which our learning algorithm, the SVM, is in general robust—in order to pick up signals in the data that may not be captured by applying an arbitrary ‘start’ and ‘end’ cut-off to the data.

### 4.3. Time-series feature extraction

Time-series data must be transformed into appropriate inputs in order to be used in supervised learning models. This process, called time-series feature extraction, involves computing summary features of the time series, which can range from simple means to complex wavelet transforms. To test the feasibility of our approach using only simple summary features, we extracted the following features within each (*t_*0*_, p*) time interval based on the findings of [31]: (i) the arithmetic mean of daily temperature and (ii) mean precipitation frequency, with the frequency defined as the time interval (in days) between peaks (local maxima) of daily precipitation. In the electronic supplementary material, we present an alternative method of feature extraction using singular value decomposition.

### 4.4. Independent model training and prediction

The goal of our independent model-building step is to identify dynamically, through the continually updating performance of a collection of models, the periods of the year that are most predictive of annual dengue outbreaks, in order to exploit a small number of them to generate forecasts.

To forecast outbreak years, we trained a collection of SVM classifiers on an initial 7 year training period and produced annual forecasts incorporating the most recently available weather information using a dynamic, 1 year expanding training window. A unique SVM was trained for each of the (*t_*0*_, p*) time intervals, resulting in a total of 432 independent models trained per year. Each model generated out-of-sample predictions for the remaining 10 years of data. Predictions were made by classifying the 30 out-of-sample data points corresponding to the weather information preceding the target year, and taking a majority vote. In order to handle highly nonlinear relationships between weather variables, both radial basis function and sigmoid kernels were used and evaluated for performance and show results for the best respective kernel in each city. We tuned model parameters (gamma, soft margin cost function and coefficient) using 10-fold cross-validation.

SVMs, a supervised learning method for classification, were used because of their flexibility in the face of complex, nonlinear decision boundaries and their robustness to overfitting and outliers. The property that underpins these advantages is known as the ‘large-margin classifier’. SVMs are also known for their good performance in high-dimensional feature space, which is advantageous for the scale-up of the model to include dozens more predictors.

### 4.5. Model selection

From the resulting collection of 432 models, the best-performing models (*n* = 11) were selected each year based on (i) historical out-of-sample prediction accuracy (per cent of outbreak forecasts correct) and (ii) out-of-sample prediction accuracy of neighbouring models (representing similar time intervals). These models thus represent strongly predictive periods of the year preceding outbreaks, and the algorithm rewards the high performance of similar temporal windows over the high performance of a time window whose neighbours exhibit poor prediction tendencies. Because the model-building process is dynamic, resulting in a new collection of models each year with continually updating performance measures, the selection of the 11 models changes from year to year.

In order to get a sense of the out-of-sample performance of the 432 models, we allowed all models to generate 4 years of out-of-sample predictions before the top 11 models were selected based on this prediction accuracy. As a result, the ensemble approach, which exploited the predictions of the top 11 models, was used for the final 6 years of out-of-sample predictions.

### 4.6. Ensemble prediction

Ensemble learning helps improve machine learning algorithms by combining the results of multiple trained predictors in order to generate a single, robust prediction. In our approach, we combine the results from the strongest-performing models, which represent the most highly predictive time periods preceding dengue outbreaks. While there is an abundance of ensembling methods in machine learning, we use a simple majority vote of the 11 models to decide a single forecast. These single forecasts were produced for the last 6 years of the 17 year dataset, representing the culmination of a prediction process that involves: 7 year initial training period, 4 year out-of-sample model calibration period and 6 year out-of-sample ensemble prediction period. Across 20 Brazilian municipalities, this scheme produced 120 municipality years of out-of-sample ensemble predictions.

### 4.7. Dengue cycles

Our weather-based ensemble approach remains agnostic to the relationship between weather patterns and dengue outbreaks, instead allowing the data to drive model selection and predictions. However, endemic transmission of dengue fever is typically distinguished by periodic outbreak cycles of around 3–4 years. These outbreak cycles are thought to occur as a result of (i) an exhaustion of susceptibles after an outbreak and (ii) short-term cross-immunity to other circulating DENV serotypes after infection [21]. Both factors result in a depletion of the population vulnerable to infection and act as barriers to subsequent outbreaks. Independent of climate variability over the years, we expect some preservation of these cycles.

Consequently, we implemented a ‘decision rule’ in the model based on the observed transitions between epidemic and non-epidemic years across 51 Brazilian municipalities meeting endemic inclusion criteria (electronic supplementary material). Across these municipalities, we computed the mean second- and third-order Markov transition probabilities, representing the probability of transition from one outbreak state (epidemic/non-epidemic) to the opposite outbreak state (non-epidemic/epidemic) after 2 and 3 consecutive years, respectively. Thus, we obtained the transition probabilities corresponding to the following 3 and 4 year cycles: 001, 110, 0001 and 1110 (0 = non-epidemic year, 1 = epidemic year). Transition probabilities were computed based only on the first 11 years of data; that is, the years preceding the six out-of-sample ensemble predictions.

Our decision rule acts as a surrogate ‘expert opinion’, overturning the ensemble prediction if the probability of a specific Markov transition to an epidemic or non-epidemic status (based on the data from previous years) exceeded the per cent of model votes (out of 11 votes). For example, if the ensemble predicts an epidemic year to succeed two epidemic years with seven votes, the corresponding ‘strength’ of that vote is 63% (7/11), which is weaker than the corresponding observed second-order transition probability for a non-epidemic year to follow two epidemic years (0.71). In this case, the model vote would be overridden to predict a non-epidemic year instead of an epidemic year.

We compared the performance of predictions based solely on weather patterns with those which incorporate additional empirical data from outbreak cycles.

## Data Availability

All data needed to evaluate the conclusions in the paper are contained in the paper and/or the electronic supplementary material. The epidemiological data used in this study are available from the Brazilian Ministry of Health. Meteorological data (MERRA-2) are available through the Global Modeling and Assimilation Office (GMAO) at NASA Goddard Space Flight Center. The yearly dengue activity binary classification of the Brazilian municipalities used in this study and meteorological data are available through https://github.com/LeonardoClemente/SupplementaryMaterialsBrazilBinary.
